# Genome-wide identification and *in-silico* characterization of phytopathogenic *Taf14* gene in *Fusarium oxysporum* fungus, and fungicide repurposing

**DOI:** 10.1371/journal.pone.0326632

**Published:** 2025-07-02

**Authors:** Md. Darun Naim, Ahmed Imtiaj, Md. Nurul Haque Mollah

**Affiliations:** 1 Bioinformatics Lab, Department of Statistics, Faculty of Science, University of Rajshahi, Rajshahi, Bangladesh; 2 Department of Botany, Faculty of Biological Sciences, University of Rajshahi, Rajshahi, Bangladesh; Osmania University, INDIA

## Abstract

**Background:**

Phytopathogenic fungi cause global agricultural damage of around 40%, for which there is an economic loss of more than $200 billion annually. *F. oxysporum* is one of the top five phytopathogenic fungi worldwide, which is extremely resistant to high temperatures and antiseptic chemicals. Therefore, it is crucial to find alternative fungicides against the *F. oxysporum* (Fo) pathogenic fungus in order to increase crop yields. The TATA box-binding protein (TBP)-associated factor 14 (*Taf14*) gene in *Botrytis cinerea* represented by (*BcTaf14*) is considered as the disease-causing gene in crops. Therefore, Taf14 protein has been recommended as the potential receptor to explore effective fungicides against the corresponding diseases in crops. However, the *Taf14* gene in *F. oxysporum* is not yet identified and characterized.

**Objectives:**

The specific objectives of this study are to (i) identify and characterize *F. oxysporum Taf14* (*FoTaf14*) gene as the *Fusarium* wilt disease causing gene in crops, and (ii) exploring *FoTaf14* gene guided candidate fungicides against the diseases in crops.

**Methods:**

This study identified FoTaf14 proteins from the whole proteome of *F. oxysporum* by using BcTaf14 as the query protein in BLASTP search. The integrated bioinformatics studies including phylogenetic tree, domain analysis, subcellular location, pathway terms, regulatory elements and expression analysis were performed in order to characterize FoTaf14 proteins. Then we explored FoTaf14 proteins guided candidate fungicides against the *Fusarium* wilt disease in crops by molecular docking analysis.

**Results:**

The phylogenetic tree has shown that FoTaf14 and Taf14 from other fungal divisions are similar. The YEATS domain has been detected by domain analysis as having a direct impact on fungal pathogenicity. The subcellular location analysis revealed that all FoTaf14s are found in the nucleus and cytoplasm, which are directly related to fungal growth and pathogenicity. The gene ontology (GO) enrichment analysis has revealed some important GO-terms that are related to plant diseases. The *cis*-regulatory network analysis of *FoTaf14* genes has shown that C-box, MYB, ABRE, SEF4, and TAAAG motifs are associated with stress, growth, and development, whereas GATA box, GT-1 motif, SEBF, HD, and W box are associated with virulence. Expression analysis has shown that *FoTaf14* genes are highly expressed in the pathogenicity, growth, and development of *F. oxysporum.* Finally, we detected FoTaf14-protein-guided three repurposed fungicides (Oxathiapiprolin, Tolfenpyrad and Amisulbrom) for the treatment against the *Fusarium* wilt disease in crops by molecular docking analysis.

**Conclusion:**

Therefore, the findings of this study might be useful resources for taking a proper treatment plan against *Fusarium* wilt disease to increase quality crop production.

## 1. Introduction

Fungal infections account for around 80% of plant diseases, which are responsible for to about 30%–40% of agricultural productivity losses [1–3]. The average yearly economic loss caused by pathogenic fungi is projected to rise above $200 billion [4]. Approximately 8,000 fungal species are responsible for almost 1,00,000 diseases in plants [5]. The *F. oxysporum* is a common, soil-dwelling fungus recognized for its variety, which affects 586 genera and more than 1,000 plant species. It causes vascular wilt in several plants, characterized by symptoms including leaf epinasty, vascular browning, progressive wilting, defoliation, stunting, and plant mortality [6]. It is one of the top five phytopathogen in the entire world which causes *Fusarium* wilt disease in about 150 different hosts [7]. This *Fusarium* wilt disease blocks the water-conducting (xylem) vessels in plant which causes plant’s death [8]. The devastating tropical race 4 (TR4) *Fusarium* wilt strain has caused massive economic damage of banana across Southeast Asia, with Indonesia losing $121 million, Taiwan $253 million, and Malaysia $14 million annually. In Mozambique, TR4 detection forced the permanent closure of a 1,500-hectare banana farm within just four years. TR4 threatens to decrease worldwide banana yields by 36 million tons by 2040, potentially causing economic losses surpassing $10 billion [9]. This *Fusarium* wilt disease damages up to 100% of lentils [10], 100% of mung beans [11], 80% loss of tomatoes [12], 90% mortality rate of cotton seedlings in the USA, 2018 [13], 70% of watermelon [14], 80% of bananas [15], 30% of jute [16,17], and 10% of strawberries [18]. *F. oxysporum* shows resistance to a compound called benzimidazole fungicides (e.g., carbendazim and thiophanate-methyl) by mutations in the *β-tubulin* gene at locations like E198A and F200Y [19]. *Fusarium* wilt in watermelon is caused by *Fusarium oxysporum* f. sp. *Niveum*, which develops resistance to triazole fungicides (e.g., tebuconazole, difenoconazole) via mutations in the *CYP51* gene, which are essential for ergosterol production of watermelon [20]. *Fusarium verticillioides* is a significant pathogen of maize exhibiting resistance to the fungicide Phenamacril [21]. Therefore, it is essential to explore alternative potential fungicide as the inhibitor of *F. oxysporum* fungus in crops.

The TATA box-binding protein (TBP)-associated factor 14 (Taf14) gene may serve as a viable target for investigating possible fungicides to suppress *F. oxysporum*, as it is connected to the mycelial growth, development, virulence, and colonization of phytopathogenic fungus [22]. The expression of *BcTaf14* gene in *B. cinerea* increases in response to abiotic stress caused by NaCl and KCl. The *BcTaf14* gene is mainly discovered within the nucleus of *B. cinerea* cells. The recognition of H3K9cr is necessary for BcTaf14-mediated control of *B. cinerea* mycelial growth as well as full virulence [22]. The *BcTaf14* gene work in chromatin remodeling and gene transcription [22] and *ScTaf14* is required for chromosome stability, normal cell growth, and the regulation of transcription in *Saccharomyces cerevisiae* (is a fungus) [23–26]. Histone acylation functions as a metabolic sensor enabling cellular adaptation to metabolic stress by altering gene expression, a process that may be disrupted by the *Taf14* gene, resulting in crop diseases [27,26]. The *Taf14* gene showed a preference for binding to lysine crotonylation (Kcr) rather than lysine acetylation (Kac), a recognition process linked to normal plant cell growth and development [28–32]. Chromatin factors, including the YEATS domain, have surfaced as potential targets for novel antifungals because of their capacity to influence DNA transcription and repair processes through interactions with post-translationally modified histones [33].

However, the *Taf14* gene in *F. oxysporum*, is not yet identified and characterized. Therefore, in this study, we considered genome-wide identification and *in-silico* characterization of phytopathogenic *Taf14* gene in *F. oxysporum* fungus. This study also attempted to explore *Taf14* gene guided repurposable fungicide as the potential inhibitors of *F. oxysporum* fungus in crops by using molecular docking analysis. The findings of this study might be potential resources to the wet-lab researchers for taking a proper treatment plan against *Fusarium* wilt disease to increase quality plant production.

## 2. Materials and methods

### 2.1. The data source and descriptions

To explore FoTaf14 protein from the *F. oxysporum* (Fo) proteome [34,35], we have considered its proteome sequences from the *National Center for Biotechnology Information* (NCBI) database [36,37] with GenBank accession number: GCF_013085055.1 (NCBI taxonomy ID: 660027, weblink: https://www.ncbi.nlm.nih.gov/datasets/genome/GCF_013085055.1/, BioSample: SAMN14572364). In this study, this genome dataset has been utilized to explore FoTaf14 proteins by BLASTP (BLAST+ 2.15.0) [38] search with the query sequence of *B. cinerea* Taf14 (BcTaf14) protein [22].

#### 2.1.1. Integrated bioinformatics analyses.

**Fig 1:**
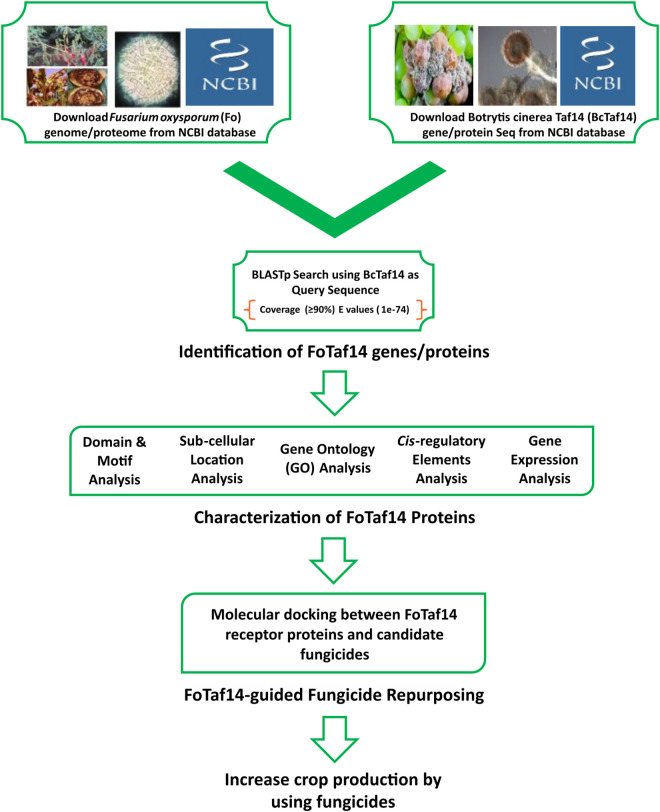
The graphical workflow of this study.

The integrated bioinformatics studies include BLASTP search, multiple sequence alignment (MSA), phylogenetic tree modeling, functional domain analysis, subcellular location, GO analysis, *cis*-acting regulatory elements (CAREs) analysis, expression analysis, homology modeling, structure prediction with *in-silico* validation and fungicide repurposing [39,40]. The detailed pipeline of this study is given in **Fig 1**.

### 2.2. Identification of *FoTaf14* gene

The basic local alignment search technique for proteins BLAST+ 2.15.0 search using BcTaf14 protein sequence as the query sequence has been used to investigate the FoTaf14 protein from *F. oxysporum* proteome of NCBI database [41]. The protein sequence of FoTaf14 has been retrieved using query coverage (≥90%) and E-values (1e-74) [42,43]. However, only the top-scorer aligned sequences have been considered as the sequences of FoTaf14 protein. The protein ID and encoded protein length of FoTaf14 proteins have been retrieved from the NCBI database. The molecular weight, pI, and GRAVY of FoTaf14 protein sequences have been calculated using ExPASy [44]. The MSAs of the expected FoTaf14 protein sequence has been constructed using the Clustal-W [45] method and the MEGA 11 version 11.0.13 [46] package. By performing phylogenetic tree analysis of FoTaf14 proteins using the Neighbor-joining technique [47], 1,000 bootstrap repetitions [48] have been utilized to confirm the evolutionary connection. The evolutionary distances have been determined using the equal input technique [49].

### 2.3. Characterization of FoTaf14 protein

#### 2.3.1. Conserved domains and motifs analysis.

The conserved domains have been retrieved from the CDD database [50]. The primary objective is to annotate proteins with domain patterns and identify protein domain structure to facilitate the identification of protein nomenclature and projected function. The TBtools-II (a Toolkit for Biologists integrating various biological data-handling tools) has been used to display the CDD’s result [51]. We have investigated the conserved motifs in all of the anticipated FoTaf14 proteins using the Multiple Expectation Maximization for Motif Elicitation (MEME), version 5.5.0 webtool [52]. The TBtools-II has been used to display the motif’s result [51].

#### 2.3.2. Sub-cellular localizations analysis.

The web-tool WoLF PSORT has been used to predict the subcellular location of the FoTaf14 proteins [53]. The web tool anticipates the subcellular localization of proteins based on their amino acid sequences. We have been using the TBtools-II program to display the result [51].

#### 2.3.3. Functional enrichment analysis of FoTaf14 protein.

The Gene Ontology (GO) analysis has been carried out using an online database COFACTOR server. CscoreGO is a scoring system on the COFACTOR server that estimates the likelihood of a protein having a specific function. Scores range from 0 to 1, with higher scores indicating greater confidence [54]. All the pathways have been analyzed by QuickGO database, version 2024-12-15 (S1 Fig in S1 Text) [55].

#### 2.3.4. *FoTaf14* gene regulatory network analysis.

CAREs analysis has been done by New PLACE database (version 30.0) [56]. This database contains motifs found in plant *cis*-acting regulatory DNA elements, based on previous publications. The TBtools-II application has been utilized to represent this data [51].

### 2.4. Expression analysis of *FoTaf14* gene

The *F. oxysporum* expression dataset is available in the NCBI GEO database, specifically under dataset GSE59311, associated with BioProject ID PRJNA255040 [57]. Comparative microarray-based gene expression data is about *Con7−1*, a common regulator of morphogenesis and pathogenicity in *F. oxysporum*. We have utilized the average of mutant types divided by the average of wild types to calculate fold change (FC) for genes that differed significantly from others. The computation of Log2-fold-change (Log2FC) use logarithm base 2 to denote the differential expression data of genes. The most widely used methods for RNA-seq or microarray gene expression analysis, Log2FC values are used [58,59]. To find differentially expressed genes between stress and control, millions of genes in genome-wide data are tested against a null hypothesis. The False Discovery Rate (FDR), which refers to the expected proportion of false positive genes within a given set of genes, has been presented as a method for determining the statistical significance of such a set [60]. This study regarded significant for p-values < 0.05 using the two-tailed student’s t-test.

### 2.5. FoTaf14 protein guided fungicide repurposing

The discovery of De-novo drugs [61–63] as well as agrochemicals [64–66] is a laborious, costly, time-consuming and extremely risky procedure, where agrochemicals (fungicides) are generally used to control pests and pathogens. On the other hand, drug (fungicide) repurposing, also known as identifying novel uses for existing fungicides, is a strategy to reduce time and expenses associated with the fungicide discovery. This is because the toxicity, pharmacokinetic properties, and biological activity of the existing fungicides are already well-established [67–71]. Therefore, in this study, we have considered FoTaf14 protein guided fungicide repurposing to inhibit *Fusarium* wilt disease in plants. Initially, the SWISS-MODEL web-tool has been utilized to design three-dimensional (3D) homology model of FoTaf14 proteins using the Uniport template of W9ZNE1, W7MJJ3 (S2 Fig in S1 Text) [72]. For the 3D modeling of FoTaf14 proteins, GMQE score >0.8 and sequence identity >90% have been considered. Ramachandran plot (Rplot) has been used for the validation of these proteins. According to Rplot, residues of these proteins have been present in most favored regions [A, B, L] ≈90% (S3 Fig in S1 Text) [73]. Candidate ligands for the FoTaf14 proteins have been explored by molecular docking analysis. This study utilized 36 approved potentials of agricultural fungicides for the repurposing [74]. The PubChem database has been used to retrieve the 3D structures of candidate drug agents [75]. The Discovery Studio Visualizer has been used to visualize the 3D structures of protein interfaces [76]. The receptor proteins have been preprocessed by removing water molecules and adding charges using AutoDock tools, version 4.2.6 [77]. The drug agents have minimized energy and preprocessed by PyRx tools [78]. Subsequently, molecular docking between receptors and ligands has been performed to calculate their binding affinity scores (kcal/mol) by using PyRx tools [78].

### 2.6. Ethics statement

This study did not involve human or animal subjects, and therefore an ethics statement is not required.

## 3. Results

### 3.1. Identification of FoTaf14 proteins

**Fig 2:**
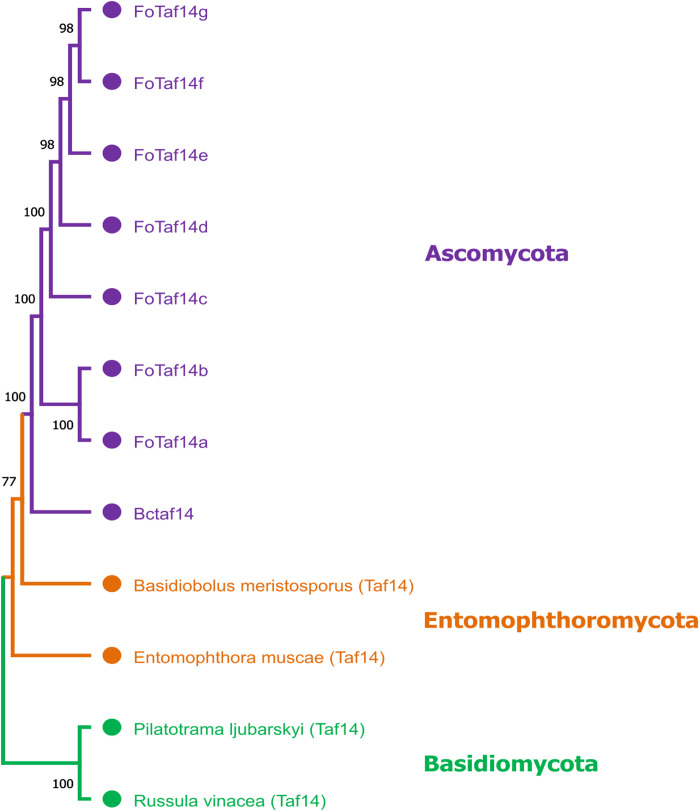
The NJ phylogenetic tree of FoTaf14 and several Taf14 proteins from different fungi have been constructed using MEGA 11. In this tree, the FoTaf14, BcTaf14 and Taf14 proteins of different fungi have been represented by purple, orange and green color, respectively. Here FoTaf14 proteins of *F. oxysporum* and *B. cinerea* have been represented by purple which denotes Ascomycota, orange color represents Entomophthoromycota (*Basidiobolus meristosporus* and *Entomophthora muscae*), and green color represents Basidiomycota (*Pilatotrama ljubarskyi* and *Russula vinosa*) division.

We have found seven FoTaf14 proteins from the *F. oxysporum* genome that are related to the BcTaf14 protein using top-scorer aligned sequences (**Table 1**). For the convenience of presentation, we have denoted to the Taf14 proteins from *F. oxysporum* as FoTaf14 and those from *B. cinerea* as BcTaf14. After multiple sequence alignments (MSAs) of seven FoTaf14 (FoTaf14a, FoTaf14b, FoTaf14c, FoTaf14d, FoTaf14e, FoTaf14f, and FoTaf14g), one BcTaf14, and four Taf14 proteins (BmTaf14, EmTaf14, PlTaf14, and RvTaf14) from other four fungi (*Basidiobolus meristosporus, Entomophthora muscae, Pilatotrama ljubarskyi* and *Russula vinosa*) a phylogenetic tree has been constructed (**Fig 2**).

**Table 1:** Basic information of predicted FoTaf14 proteins of *F. oxysporum* (Fo).

IDs	Protein ID	Length (aa)	M.W. (D)	pI	GRAVY	Aliphatic index
FoTaf14a	XP_018249795.1	233	27079.51	6.2	−0.844	69.83
FoTaf14b	XP_018249798.1	214	24989.09	5.33	−0.821	70.98
FoTaf14c	XP_018249797.1	226	26156.56	5.26	−0.731	77.57
FoTaf14d	XP_018249794.1	245	28246.98	6.12	−0.76	75.96
FoTaf14e	XP_018249796.1	232	26826.33	5.18	−0.707	79.74
FoTaf14f	XP_018249799.1	200	23184.02	5.13	−0.812	74.05
FoTaf14g	XP_018249793.1	251	28916.76	5.94	−0.737	78.01

N.B: The protein ID and protein length (aa) have been collected from NCBI database. The molecular weight, isoelectric point (pI), and grand average of hydropathicity (GRAVY), Aliphatic index values have been collected from the ExPASy. Molecular weights (M. W.) have been measured in Daltons (D) and “aa” means amino acid.

We observed that FoTaf14 proteins are closer to BcTaf14 compared to BmTaf14, EmTaf14, PlTaf14, and RvTaf14 proteins. Among the FoTaf14 proteins, FoTaf14a, FoTaf14b and FoTaf14c are much closer to BcTaf14 protein. *B. meristosporus* and *E. muscae* fungi represent Entomophthoromycota division. Additionally, *P. ljubarskyi* and *R. vinosa* fungi represent the Basidiomycota division. The key finding is different Taf14s form different cluster such as BcTaf14 and FoTaf14s belongs to Ascomycota division, BmTaf14 and EmTaf14 belongs to Entomophthoromycota division, and PlTaf14 and RvTaf14 belongs to Basidiomycota division of fungi.

The lengths of FoTaf14 proteins vary from 200 amino acids (FoTaf14f) to 251 amino acids (FoTaf14g) (**Table 1**). The isoelectric point (pI) values of the FoTaf14 genes indicated a mild acidity. The FoTaf14 proteins exhibit a grand average hydropathicity index (GRAVY) or peptides hydrophobicity values vary from −0.707 (FoTaf14e) to −0.844 (FoTaf14a). The aliphatic index of FoTaf14 proteins vary from 69.83 (FoTaf14a) to 79.74 (FoTaf14e).

### 3.2. Characterization of FoTaf14 proteins with respect to BcTaf14 protein

#### 3.2.1. Conserved domains and motifs analysis.

**Fig 3:**
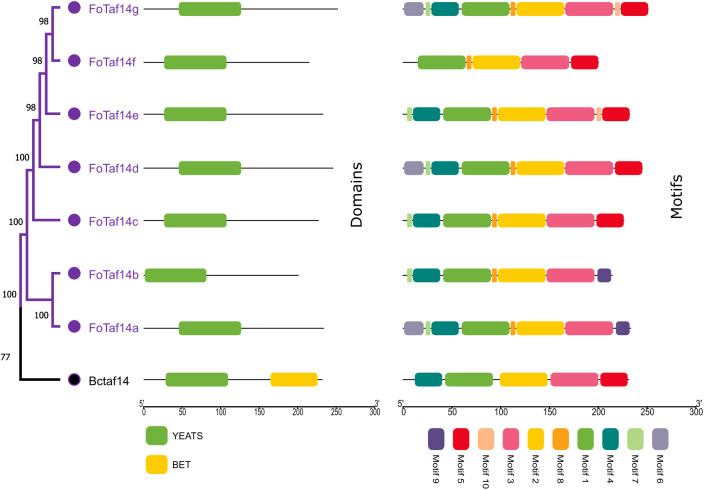
The conserved domains and motifs of the BcTaf14 and FoTaf14 proteins have been drawn by using CDD database information. Different colors represent different conserved domains and motifs.

This study compares the conserved domains of FoTaf14 with those of BcTaf14 proteins. Fig 3, reveals that both FoTaf14s and BcTaf14 exhibit nearly identical conserved domains, including the YEATS domain. We have identified 10 significant motifs for FoTaf14 and BcTaf14 proteins by using MEME-suite analysis. The majority of the FoTaf14 proteins exhibit comparable motif distributions, ranging from 7 to 9. Only BcTaf14 and FoTaf14f have displayed five motifs. The annotated conserved motifs 1 represents YEATS domain. The key finding is we have found YEATS domain and motifs 1 represent the domain.

#### 3.2.2. Sub-cellular localizations.

**Fig 4:**
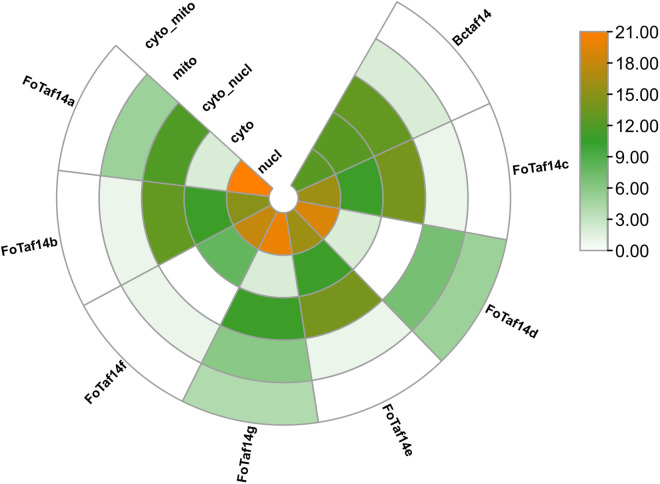
Sub-cellular localization of BcTaf14 and FoTaf14 proteins. The numbers roughly indicate the number of nearest neighbors to the query predicted protein sequences which localize to each site. N.B. nucl – nucleus, cyto – cytosol, and mito – mitochondria.

Subcellular localization study has been carried out to learn more about the FoTaf14 proteins cellular presence. The majority of FoTaf14s and BcTaf14 protein have been found in the nucleus and cytosol, according to the study (**Fig 4**). Some of the FoTaf14 proteins have also been found in mitochondria (**Fig 4**).

#### 3.2.3. Enrichment analysis with GO-terms and pathways.

The GO enrichment analysis has been conducted to examine the association of putative FoTaf14 proteins with BPs and MFs. The study has identified numerous significant BPs and MFs that are closely associated with cell growth and development (Table 2). For example, all the FoTaf14 and BcTaf14 proteins have been engaged with BP terms “cellular process”, “RNA biosynthetic process”, “transcription, DNA-templated”, “cellular macromolecule metabolic process”, “primary metabolic process”, “biological regulation”, “regulation of biological process”, “histone acetylation” (Table 2). We observed that all FoTaf14 genes are significantly involved with the MFs terms as “catalytic activity”. Only the BcTaf14 and FoTaf14f proteins have been found to be involved with the MF terms as “heterocyclic compound binding”, “organic cyclic compound binding”, and “ATP binding”, pathways (S1 Fig in S1 Text).

**Table 2:** Enriched GO-terms (Biological processes and Molecular functions) corresponding to the BcTaf14 and predicted FoTaf14 proteins analyzed through COFACTOR web-based tools.

Biological processes (BPs)		Molecular functions (MFs)	
GO IDs	Description with CscoreGO	GO IDs	Description with CscoreGO
GO:0009987	cellular process (1.00)	GO:1901363	heterocyclic compound binding (1.00)
GO:0032774	RNA biosynthetic process (0.99)	GO:0097159	organic cyclic compound binding (1.00)
GO:0006351	transcription, DNA-templated (0.98)	GO:0005524	ATP binding (0.97)
GO:0044260	cellular macromolecule metabolic process (0.83)	GO:0003824	catalytic activity (0.67)
GO:0044238	primary metabolic process (0.82)		
GO:0065007	biological regulation (0.75)		
GO:0050789	regulation of biological process (0.75)		
GO:0016573	histone acetylation (0.59)		

#### 3.2.4. CAREs analysis.

**Fig 5:**
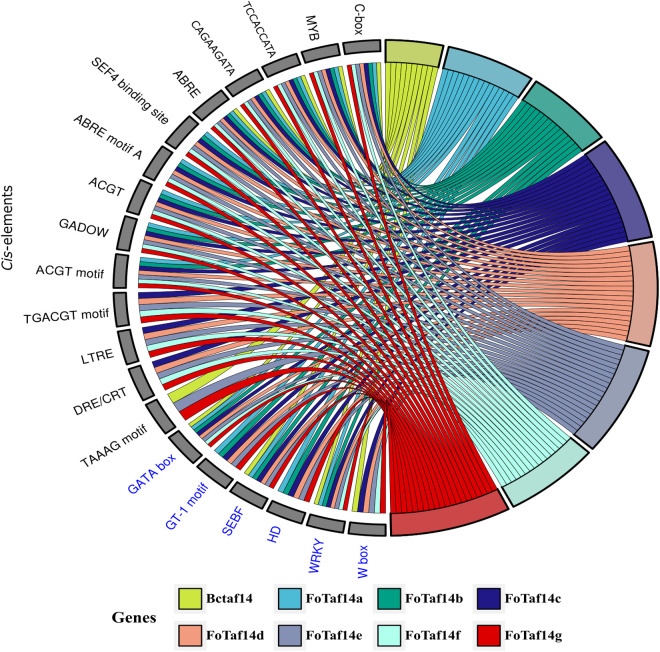
The *cis*-acting regulatory elements of *BcTaf14* and predicted *FoTaf14* genes. The black color represents growth and development, and the blue color represents virulence related *cis*-elements.

The CAREs study has been performed to investigate how those regulators regulate *FoTaf14* genes. The findings indicate that the regulatory *cis*-elements of *FoTaf14* genes involves with virulence, and growth and development (**Fig 5**). The virulence associated *cis*-elements are GATA, GT1, SEBF, HD, WRKY, and WBOX. Growth & development associated *cis*-elements are C-box, MYB, TCCACCATA, CAGAAGATA, ABRE, SEF4, ABRE motif A, GADOW, ACGT motif, TGACGT motif, LTRE, DRE/CRT, and TAAAG motif.

### 3.3. Gene expression analysis of *FoTaf14* genes

**Fig 6:**
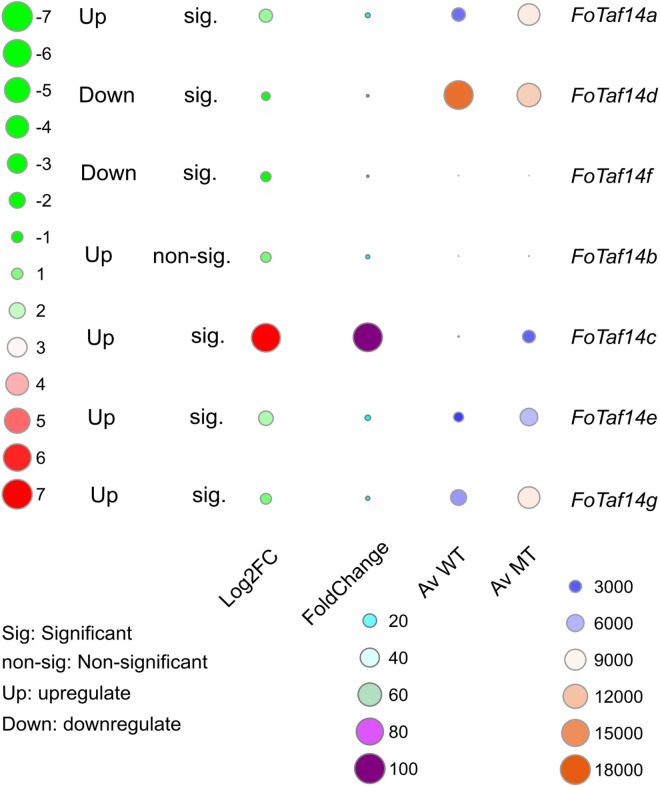
Analysis of the *FoTaf14* genes expression level on wild types and mutant types. Wild-type and mutant type samples are represented by orange to blue color. FC values of samples are represented by purple to past color. Log2FC values of samples are represented by red to light color.

A computational analysis gene expression study has been performed to verify the biological existence of the *FoTaf14* genes in *F. oxysporum*. The Log2FC values of the sample data have been used to determine whether the expressions of *FoTaf14* genes are upregulated or downregulated. A comparative investigation of gene expression was conducted between the wild type and *Con7−1* mutant type of *F. oxysporum*, where the mutant type has been shown to increase pathogenicity. An *in-silico* analysis, based on the expression data, has revealed that the majority of *FoTaf14* genes have higher expression in the mutant type, with the exceptions of *FoTaf14d* and *FoTaf14f* (**Fig 6**). The study has revealed that the expression of the majority of *FoTaf14* genes is downregulated in the wild type, with the exception of *FoTaf14d* and *FoTaf14f*. All *FoTaf14* genes exhibit significant expression in mutant types except *FoTaf14b* gene. The key findings are most of the *FoTaf14* genes are pathogenicity causal genes.

### 3.4. FoTaf14 protein guided fungicide repurposing

To investigate repurposable fungicides for the inhibition of the pathogenic fungus *F. oxysporum* on crops by molecular docking analysis, the 3D-structure (S2 Fig in S1 Text) of the FoTaf14 proteins have been obtained from SWISS-MODEL, utilizing the UniProt templates W9ZNE1 and W7MJJ3, which have been confirmed using the Ramachandran plot (S3 Fig in S1 Text). We performed molecular docking studies to calculate the binding affinity scores (BAS) between proposed receptors (FoTaf14s) and approved fungicides from the literature review. To find the most promising fungicides candidate for treating *Fusarium*-wilt, molecular docking (in kcal/mol) has been performed to evaluate the 36 approved fungicides for various fungal infections. Seven FoTaf14 proteins have been utilized as receptors. BIOVIA Discovery Studio has been employed to eliminate heteroatoms, water molecules, and unnecessary components. Protein energy minimization has been conducted via PyRx software. Each protein is subsequently converted into the appropriate PDBQT format via AutoDock tools.

**Fig 7:**
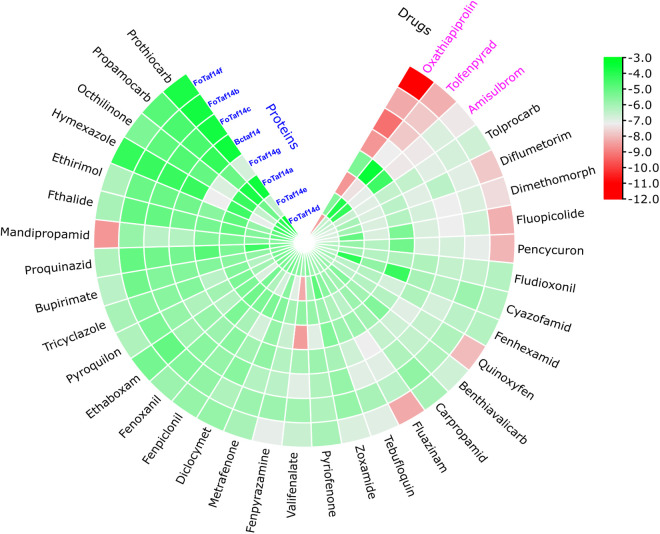
Molecular docking analysis between predicted FoTaf14 proteins and 36 approved fungicides as target-ligand. Visualization of top-scorer target-ligand complexes, with blue color text representing the proteins, black color text indicating the fungicide candidates and pink color representing the top 3 potential fungicides. Green to red represents the docking score degree and the lower to higher binding affinity.

The BASs have been organized in matrix A = (Aij) in a descending order, with receptor proteins listed in rows and ligands in columns. Consequently, the three highest-ranked fungicides (Oxathiapiprolin, Tolfenpyrad, and Amisulbrom) may be promising candidates for agriculture. The compounds exhibit average BAS of −11.0, −7.8, and −7.3 kcal/mol, respectively, against seven FoTaf14 receptor proteins (**Fig 7**). The top 3 fungicides have a BAS of less than −7.0 kcal/mol against FoTaf14 receptor proteins.

**Fig 8:**
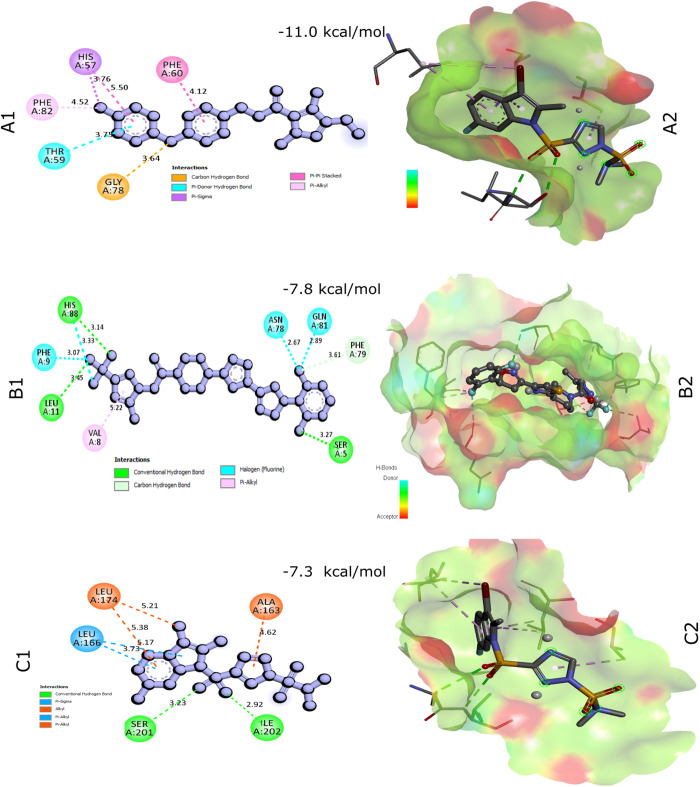
Interactions between the top three receptor protein and potential fungicide candidates binding affinity score matrices have been displayed. (A1-A2) Surface of Fotaf14f_oxathiapiprolin interaction in 2D & 3D. (B1-B2) Surface of FoTaf14b_tolfenpyrad interaction in 2D & 3D. (C1-C2) Surface of FoTaf14c_amisulbrom interaction in 2D & 3D.

**Fig 8** (A1 and A2) displayed the 2D and 3D surface view of Fotaf14f_oxathiapiprolin interaction respectively, The 2D and 3D surface view of FoTaf14b_tolfenpyrad interaction were displayed in Fig. 8(B1 and B2), respectively, and FoTaf14c_amisulbrom interaction in Fig. 8(C1 and C2), respectively. Furthermore, the toxicological and pharmacokinetic features of the compound have been evaluated to determine its effectiveness and indemnification level.

## 4. Discussion

Phytopathogenic fungi are significant risk factors for agriculture, resulting in yearly economic losses exceeding $200 billion [4]. *F. oxysporum* (fungus) is the top-5th phytopathogenic fungus worldwide [79–81]. Consequently, it is essential to identify an efficient fungicide to suppress *F. oxysporum* to enhance crops yield production. The *Taf14* gene may serve as a promising target for developing potential fungicides that inhibit *F. oxysporum*, as it is linked to mycelial proliferation, development, virulence, and colonization in phytopathogenic fungi [22–26]. We have identified seven FoTaf14 proteins from the *F. oxysporum* proteome by utilizing the BcTaf14 protein as a query sequence by BLASTp search based on MSAs, as both *F. oxysporum* and *B. cinerea* are classified under the same Ascomycota division (**Fig 2**) [22]. To computationally validate these seven FoTaf14 proteins as disease-causing entities, we have analyzed their characteristics, including conserved domains and motifs, subcellular localization, gene ontology (GO), gene regulatory factors, and expression patterns in relation to the BcTaf14 protein, and repurposed it with other fungicides (**Fig 1**) as shown below.

The FoTaf14 protein may modulate pathogenicity-related proteins, hence affecting the virulence of *F. oxysporum*, similar to the role of BcTaf14 protein in *B. cinerea* [22]. The anticipated FoTaf14 proteins exhibits a comparable YEATS domain to that of the BcTaf14 protein (**Fig 3**). The YEATS domain of *F. oxysporum* may be crucial to virulence, regulating gene expression, conferring stress resistance, and facilitating invasive growth, analogous to its function in *C. albicans* [22,82,83]. Analogous to *BcTaf14*, *FoTaf14* genes may serve as a transcription-associated factor and a component of many chromatin-associated complexes; thus, nuclear localization is crucial for its functionality [22,84]. The majority of FoTaf14s identified the nucleus of *F. oxysporum*’s cell, where it may influence the fungus’s pathogenicity according to analogous functions in *B. cinerea* [22]. Fungal growth-related processes, including glycolysis, the pentose phosphate pathway, and fatty acid synthesis, take place in the cytoplasm alongside all FoTaf14s present therein [85]. The majority of the anticipated FoTaf14 protein has been significantly localized in the nucleus, cytosol, and mitochondria (**Fig 4**), with the first two sites recognized for their role in gene regulation mechanisms [86,87]. The GO enrichment analysis of the FoTaf14 protein has identified several critical BP and MF terms that are directly associated with virulence, growth, and development, which closely resemble those of the BcTaf14 protein (**Table 2**). The dysregulation of “histone acetylation” in *F. graminearum* (which could also be relevant to *F. oxysporum*) results in the suppression of fungal growth, pathogenicity, and mycotoxin synthesis [88,89]. The “ATP-Binding” gene (*FgABCC9*) in *F. graminearum* is crucial for the fungal transport of salicylic acid, pathogenicity, resistance to fungicides, and mycelial growth in wheat [90]. ATP-binding cassette transporters, involved in ATP-binding pathways, may significantly contribute to the pathogenicity of *F. oxysporum* on host plants by providing resistance against plants defense [91]. In pathogenic fungi such as *F. oxysporum*, “histone acetylation” regulates virulence by influencing the expression of pathogenicity-related genes [92,93]. Hydrocarbons, carbohydrates, lipids, amino acids, proteins, nucleic acids, and organic acids are some of the most important metabolic products. These compounds are produced by major “metabolic pathways”, encompassing amino acid and protein synthesis, as well as nucleotide and nucleic acid synthesis, and may be fundamental to the virulence, growth, and development *of F. oxysporum* [94,95].

The WRKY and W box regulates plant defense genes against fungal infections, suggesting that these *cis*-elements may perform similar roles in *F. oxysporum* (**Fig 5**) [96–98]. MYB1, is also found in *FoTaf14* genes, regulates the formation of hypha-derived appressoria in *Magnaporthe oryzae*, which are specialized structures for infection, and is crucial for full pathogenicity on rice leaves [99]. ABRE is implicated in the growth, development, and virulence of the pathogenic fungus *Stagonospora nodorum*, which might have analogous activities in *F. oxysporum* [100]. The expression analysis has revealed that *FoTaf14* genes are highly expressed in the pathogenicity, growth, and development of *F. oxysporum* [57]. The expression of *FoTaf14* genes, like *BcTaf14*, may be upregulated by abiotic and thermal stresses (**Fig 6**) [22]. The features of FoTaf14 genes have been revealed, together with their pathogenicity and virulent activity.

Consequently, FoTaf14-directed Oxathiapiprolin, Tolfenpyrad, and Amisulbrom may serve as promising candidates for a novel fungicide, according to their resemblance to heterocyclic compounds and their single-ring structure, which is analogous to the approved fungicides Halofuginone and Kaempferol in fighting fungal pathogens (**Figs 7 and 8**) [101,102]. Oxathiapiprolin demonstrates high inhibitory efficacy against certain agriculturally significant plant-pathogenic oomycetes, such as *Phytophthora* spp., *Pseudoperonospora cubensis*, *Plasmopara viticola*, *Pythium ultimum*, *Peronophythora litchii*, and *Peronospora parasitica* [103]. Tolfenpyrad significantly suppresses spore germination, with studies indicating up to 85.58% suppression of powdery mildew (*Leveillula taurica*) in chillies at a recommended dose of 150 g active ingredient/hectare [104]. Amisulbrom 20% decreased the virulence of potato Late blight disease (*Phytophthora infestans*) up to 92.1% increased potato yields to 30.25–32.50 ton/hectare when applied at a rate of 500 mL/hectare [105].

## 5. Conclusion

The *Taf14* gene regulates the growth and virulence of phytopathogenic fungus, so it is regarded as a pathogenic gene. Therefore, this *in-silico* study predicted *FoTaf14* genes as *Fusarium* wilt disease-causing genes and proposed it as a potential target for the development of effective fungicides against the disease. This study identified seven *FoTaf14* genes in *F. oxysporum* and analyzed them compared to the *BcTaf14* gene utilizing various bioinformatics tools and databases. Following the *in-silico* conformation of FoTaf14 as disease-causing proteins, molecular docking study has been conducted for possible candidates of repurposable fungicides against phytopathogenic fungi. We detected top-ranked 3 prospective repurposable fungicides (Oxathiapiprolin, Tolfenpyrad, and Amisulbrom) as the treatment against *Fusarium* wilt disease in crops. Although this analysis is computer-based, the study’s findings require experimental validation for developing an effective treatment strategy utilizing those potential fungicides against *Fusarium* wilt disease to improve crop quality production.

## Supporting information

S1 Text **S1 Fig.** GO pathways of BcTaf14 and projected FoTaf14 proteins. The color sequence Pink (0.4–0.49) <Past (0.5–0.59) <Green (0.6–0.69) <Yellow (0.7–0.79) <Orange (0.8–0.89) <Red (0.9–1.0) represents the lower to higher scores. **S2 Fig.** 3D structure of BcTaf14 and predicted FoTaf14 proteins. The SWISS-MODEL web tool is employed to predict 3D protein models with a GMQE score of at least 0.8 and a sequence identity of at least 90%. **S3 Fig.** Ramachandran plot of BcTaf14 and projected FoTaf14 proteins for the validation of their 3D models. Residues of these proteins have been present in most favored regions [A, B, L] ≈90%. **S1 File.** *FoTaf14* gene expression analysis data.
(ZIP)
