# Supplementary figures and images for: Genome-wide identification and in-silico characterization of phytopathogenic Taf14 gene in Fusarium oxysporum fungus, and fungicide repurposing
Source: PLoS One. 2025 Jul 2;20(7):e0326632. doi: 10.1371/journal.pone.0326632 (PMC12221007; doi:10.1371/journal.pone.0326632)

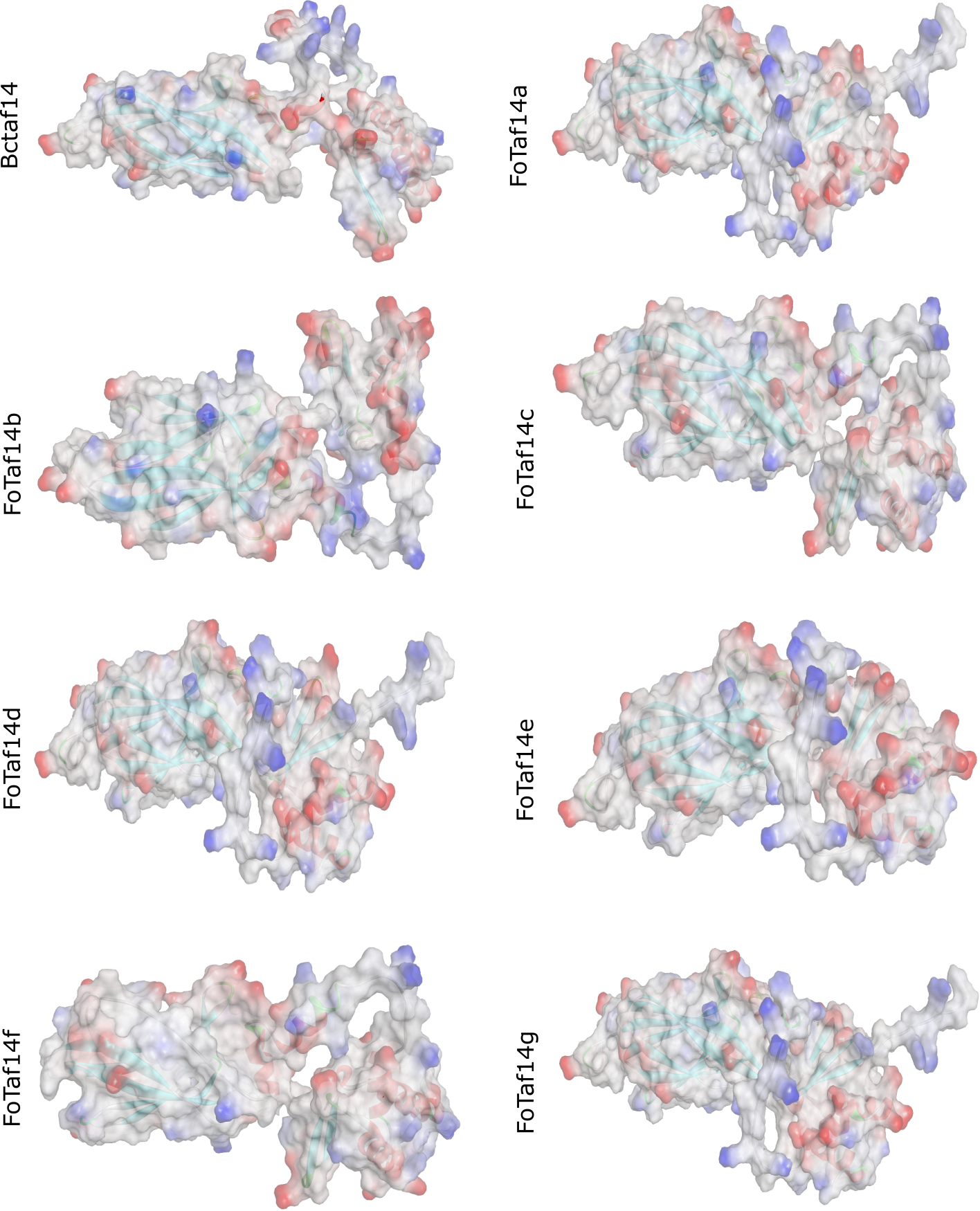

Supplement: S1 Text — S1 Fig. GO pathways of BcTaf14 and projected FoTaf14 proteins. The color sequence Pink (0.4–0.49) <Past (0.5–0.59) <Green (0.6–0.69) <Yellow (0.7–0.79) <Orange (0.8–0.89) <Red (0.9–1.0) represents the lower to higher scores. S2 Fig. 3D structure of BcTaf14 and predicted FoTaf14 proteins. The SWISS-MODEL web tool is employed to predict 3D protein models with a GMQE score of at least 0.8 and a sequence identity of at least 90%. S3 Fig. Ramachandran plot of BcTaf14 and projected FoTaf14 proteins for the validation of their 3D models. Residues of these proteins have been present in most favored regions [A, B, L] ≈90%. S1 File. FoTaf14 gene expression analysis data. (ZIP) [file pone.0326632.s001.zip › Supplementary_Figure_2.tiff]

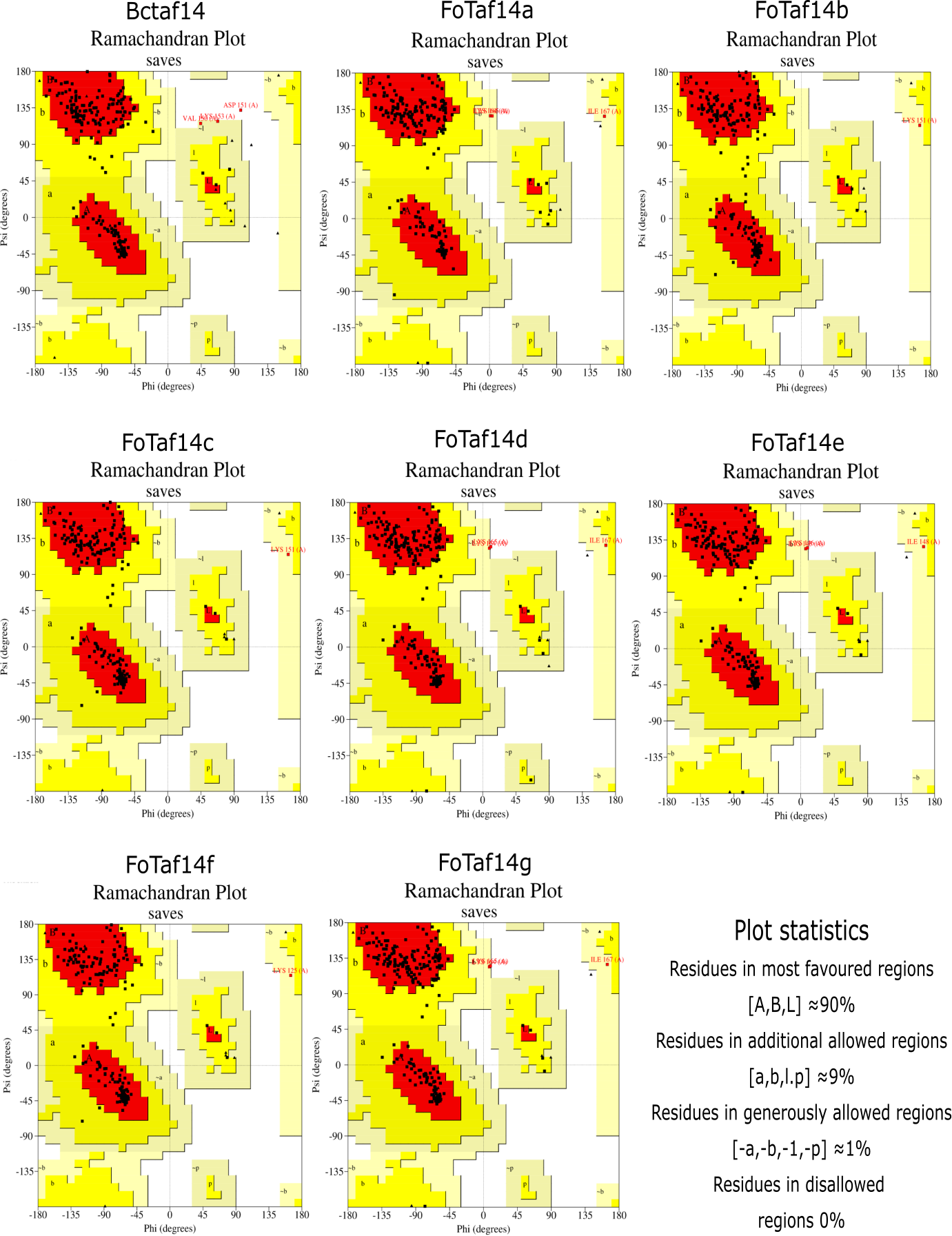

Supplement: S1 Text — S1 Fig. GO pathways of BcTaf14 and projected FoTaf14 proteins. The color sequence Pink (0.4–0.49) <Past (0.5–0.59) <Green (0.6–0.69) <Yellow (0.7–0.79) <Orange (0.8–0.89) <Red (0.9–1.0) represents the lower to higher scores. S2 Fig. 3D structure of BcTaf14 and predicted FoTaf14 proteins. The SWISS-MODEL web tool is employed to predict 3D protein models with a GMQE score of at least 0.8 and a sequence identity of at least 90%. S3 Fig. Ramachandran plot of BcTaf14 and projected FoTaf14 proteins for the validation of their 3D models. Residues of these proteins have been present in most favored regions [A, B, L] ≈90%. S1 File. FoTaf14 gene expression analysis data. (ZIP) [file pone.0326632.s001.zip › Supplementary_Figure_3.tiff]

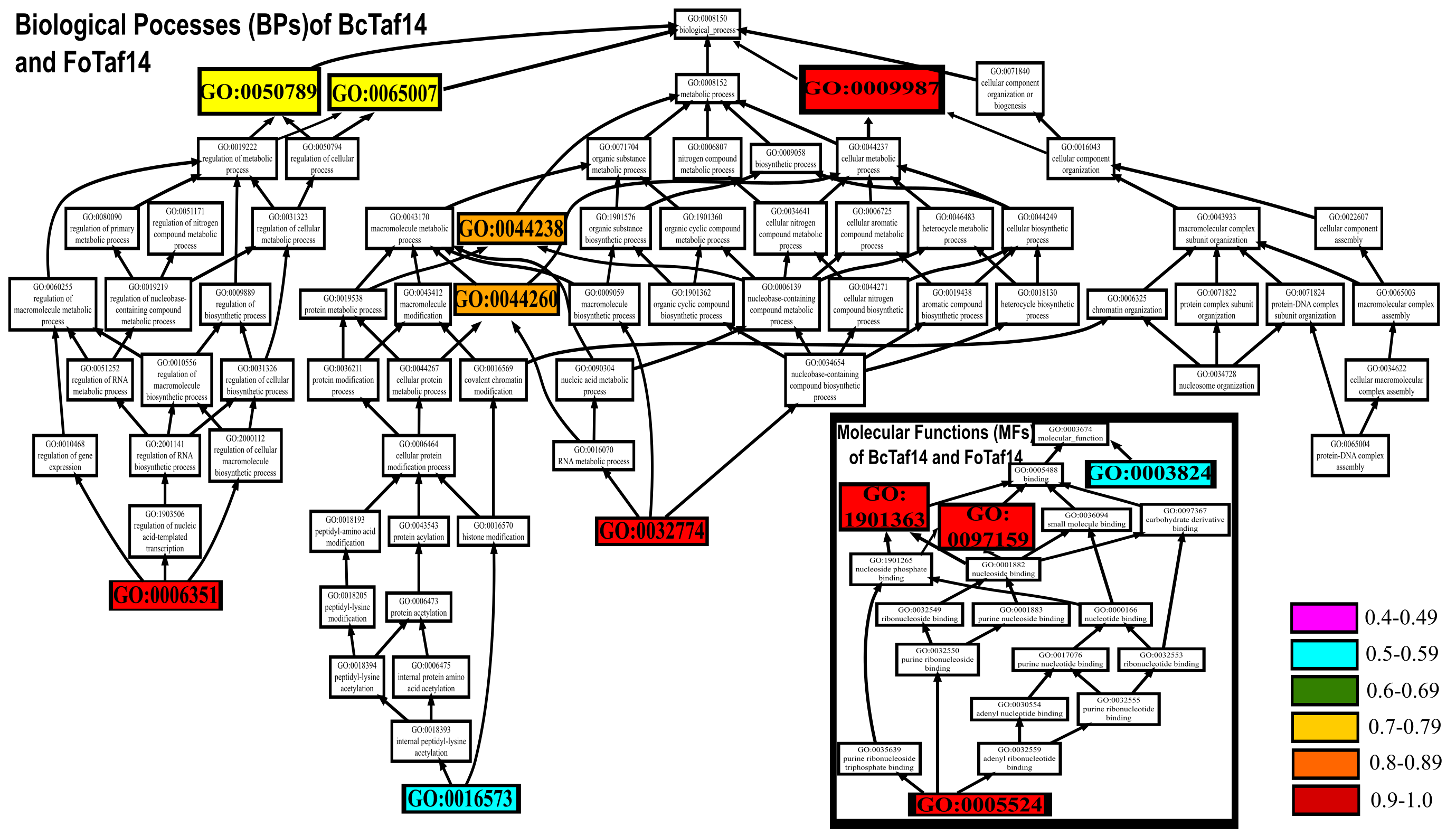

Supplement: S1 Text — S1 Fig. GO pathways of BcTaf14 and projected FoTaf14 proteins. The color sequence Pink (0.4–0.49) <Past (0.5–0.59) <Green (0.6–0.69) <Yellow (0.7–0.79) <Orange (0.8–0.89) <Red (0.9–1.0) represents the lower to higher scores. S2 Fig. 3D structure of BcTaf14 and predicted FoTaf14 proteins. The SWISS-MODEL web tool is employed to predict 3D protein models with a GMQE score of at least 0.8 and a sequence identity of at least 90%. S3 Fig. Ramachandran plot of BcTaf14 and projected FoTaf14 proteins for the validation of their 3D models. Residues of these proteins have been present in most favored regions [A, B, L] ≈90%. S1 File. FoTaf14 gene expression analysis data. (ZIP) [file pone.0326632.s001.zip › Supplementary_Figure_1.tiff]
